# Phylogenetic and functional characterization of water bears (Tardigrada) tubulins

**DOI:** 10.1038/s41598-023-31992-z

**Published:** 2023-03-30

**Authors:** Kamila Novotná Floriančičová, Athanasios Baltzis, Jiří Smejkal, Michaela Czerneková, Łukasz Kaczmarek, Jan Malý, Cedric Notredame, Stanislav Vinopal

**Affiliations:** 1 Department of Biology, Faculty of Science, J. E. Purkyně University (UJEP), Usti Nad Labem, Czech Republic; 2 Centre for Genomic Regulation, Barcelona, Spain; 3 Centre for Nanotechnology and Biotechnology, Faculty of Science, UJEP, Usti Nad Labem, Czech Republic; 4 Department of Animal Taxonomy and Ecology, Adam Mickiewicz University in Poznań, Poznań, Poland; 5 Universitat Pompeu Fabra (UPF), Barcelona, Spain

**Keywords:** Cell biology, Computational biology and bioinformatics, Molecular biology

## Abstract

Tardigrades are microscopic ecdysozoans that can withstand extreme environmental conditions. Several tardigrade species undergo reversible morphological transformations and enter into cryptobiosis, which helps them to survive periods of unfavorable environmental conditions. However, the underlying molecular mechanisms of cryptobiosis are mostly unknown. Tubulins are evolutionarily conserved components of the microtubule cytoskeleton that are crucial in many cellular processes. We hypothesize that microtubules are necessary for the morphological changes associated with successful cryptobiosis. The molecular composition of the microtubule cytoskeleton in tardigrades is unknown. Therefore, we analyzed and characterized tardigrade tubulins and identified 79 tardigrade tubulin sequences in eight taxa. We found three α-, seven β-, one γ-, and one ε-tubulin isoform. To verify in silico identified tardigrade tubulins, we also isolated and sequenced nine out of ten predicted *Hypsibius exemplaris* tubulins. All tardigrade tubulins were localized as expected when overexpressed in mammalian cultured cells: to the microtubules or to the centrosomes. The presence of a functional ε-tubulin, clearly localized to centrioles, is attractive from a phylogenetic point of view. Although the phylogenetically close Nematoda lost their δ- and ε-tubulins, some groups of Arthropoda still possess them. Thus, our data support the current placement of tardigrades into the Panarthropoda clade.

## Introduction

Tardigrades, also called water bears, are microscopic ecdysozoans belonging to the phylum Tardigrada, divided into three classes: well-defined Heterotardigrada, Eutardigrada, and dubious Mesotardigrada^1^. Tardigrades are known for their unique ability to withstand extreme environmental conditions, such as desiccation, vacuum, low/high temperatures and radiation. Several tardigrade species can undergo reversible morphological transformations into so-called tuns^2^. Tuns are compacted tardigrade bodies with legs withdrawn inward into the body cavity^3–5^. In their tun form, tardigrades enter into cryptobiosis, a state with a barely detectable metabolism^6^. Cryptobiosis helps tardigrades survive unfavorable environmental conditions, such as those listed above^6–13^. After the return of favorable external conditions, tardigrades undergo a reverse morphological change and enter the active stage of life^11,14^. However, underlying molecular mechanisms governing cryptobiosis have only begun to emerge^15–19^.

The phylogenetic position of the phylum Tardigrada is under discussion. Some authors proposed that tardigrades belong to the animal clade Panarthropoda (euarthropods and onychophorans)^20–23^, while others suggested that Tardigrada and Nematoda have a closer phylogenetic relationship than Tardigrada and Arthropoda do^24^. Despite mounting molecular evidence, the phylogenetic position of the phylum Tardigrada has remained uncertain.

The cytoskeleton provides a system for cellular movement and shape changes. It consists of three prominent protein filament families—actin, intermediate filaments and microtubules. Although each cytoskeletal filament type has different mechanical properties, dynamics, and biological roles, the systems are interrelated^25^.

Interestingly, Panarthropoda generally possess microtubules and actin filaments but lack cytoplasmic intermediate filaments (IFs)^26^. The presence of an exoskeleton providing mechanical support might cause the loss of cytoplasmic IFs in the arthropod lineage^26,27^. In tardigrades, the cytoplasmatic role of IFs was substituted by a novel lamin-derived protein Cytotardin^28^. Cytotardin does not form scaffold-like structures throughout the cell but belt-like filaments^28^. It was found in the epidermis and tissues exposed to mechanical stress and might help resist extreme conditions^28^.

Microtubules are hollow cylinders composed of evolutionarily conserved heterodimers of tubulin proteins and are critical for many cellular processes^29^. Tardigrade microtubules were observed in an electron microscopy study in claw glands^30^ and also in recent studies on tardigrade nervous system^21,31–37^.

We hypothesize that microtubules might play an essential role in tardigrade physiology and are necessary for morphological changes associated with cryptobiosis. The molecular composition of the microtubule cytoskeleton in tardigrades is unknown. Therefore, we decided to start elucidating this outstanding question by analyzing and characterizing tardigrade tubulins.

## Results

To study the molecular composition of the tardigrade microtubule cytoskeleton, we identified and analyzed tardigrade tubulin coding sequences (CDS). We used published transcriptomes and genomes—with annotated predicted CDS—of eight tardigrade taxa (Table 1). We generated three putative tardigrade tubulin homologs datasets based on full-length sequences and tubulin domains (UJEP dataset, CRG dataset, CRG tubulin domains dataset; see Methods). Next, we ran regressive T-coffee^38^ alignments of found tardigrade tubulin protein sequences with a large dataset published by Findeisen et al.^39^ containing 3524 tubulin or tubulin-like sequences from 504 species (Supplementary Data 1).

**Table 1:** List of tardigrade transcriptomes and genomes used in this study.

Species	Data type	Abbreviation	Reference/GenBank Accession number
*Echiniscoides* cf. *sigismundi*	Transcriptome	tEs	Kamilari et al.^61^
*Richtersius* cf. *coronifer*	Transcriptome	tRc	Kamilari et al.^61^
*Echiniscus testudo*	Transcriptome	tEt	Mapalo et al.^62^
*Mesobiotus philippinicus*	Transcriptome	tMp	Mapalo et al.^62^
*Milnesium tardigradum* (now *Milnesium inceptum*)	Transcriptome	tMt	GFGZ00000000.1
*Paramacrobiotus richtersi*	Transcriptome	tPr	GFGY00000000.1
*Hypsibius exemplaris*	Genome/annotated CDS	tHe	Yoshida et al.^24^ MTYJ00000000.1
*Ramazzottius varieornatus*	Genome/annotated CDS	tRv	Hashimoto et al.^51^ BDGG00000000.1

**Figure 1:**
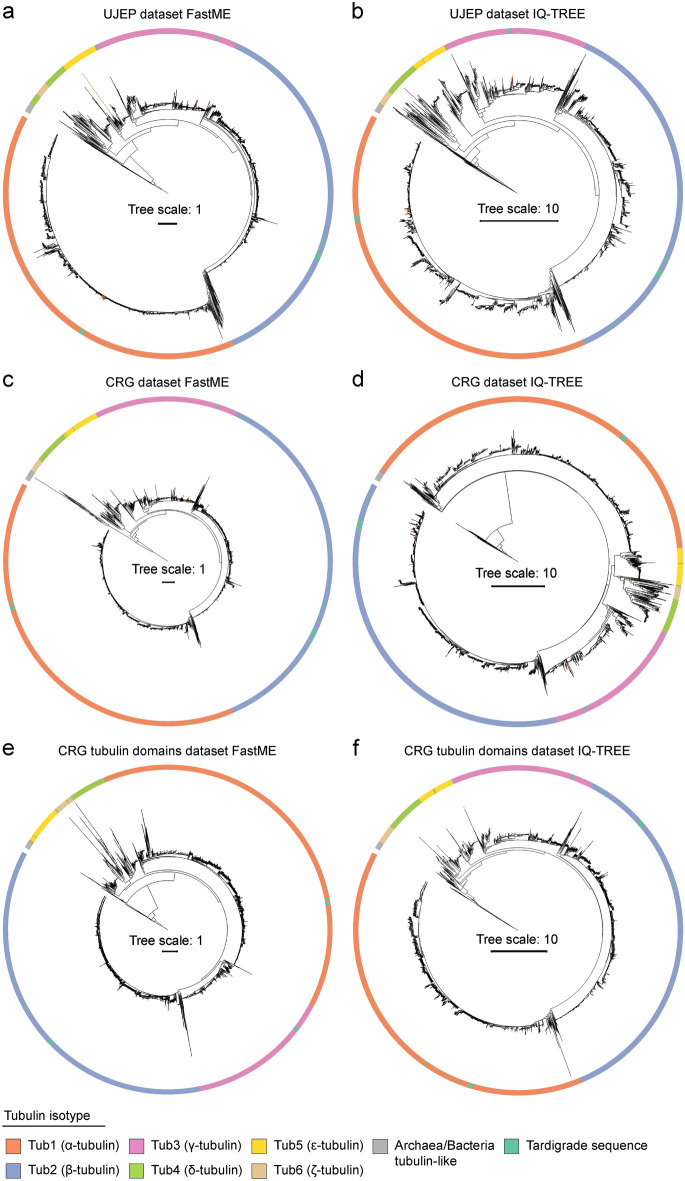
Phylogenetic analysis of tubulin homologs found in tardigrades. (**a-f**) Phylogenetic trees computed from alignments of tardigrade tubulin homologous sequences with Findeisen et al.^39^ dataset. (**a, c, e**) Minimum evolution method (FastMe). (**b, d, f**) Maximum likelihood method (IQ-TREE). (**a, b**) UJEP dataset—full tubulin sequences, (**c, d**) CRG dataset—full tubulin sequences, (**e–f**) CRG dataset—extracted tubulin domains only. All phylogenetic trees are rooted by an archaeal tubulin-like group. The legend describes color coding. See also Supplementary data 1 and Supplementary Data 2.

Next, we constructed phylogenetic trees based on the alignments to estimate which tubulin isotypes and isoforms were found in tardigrades. We used maximum likelihood (IQ-TREE) and minimum evolution (FastME) methods. All phylogenetic trees were always rooted using Archaea tubulin-like sequences, not bacterial tubulin homologs (e.g., FtsZ), to minimize the likelihood of homoplasy events due to a larger phylogenetic distance. The tardigrade tubulins we found clustered into α-, β-, γ-, and ε-tubulin isotypes (Fig. 1, Supplementary Data 2; for the unification of nomenclature, see Supplementary Table S1). Notably, we adopted simplified tubulin naming from Findeisen et al.^39^, where numbers substituted Greek letters: α-tubulin is Tub1, β-tubulin is Tub2, γ-tubulin is Tub3, δ-tubulin is Tub4, ε-tubulin is Tub5, and ζ-tubulin is Tub6. Individual tubulin isoforms are further distinguished by additional letters and numbers, such as Tub2D3 and Tub2D4, two different tardigrade β-tubulin isoforms.

This analysis allowed us to identify several divergent non-tubulin (tPr-orf74 and tPr-GFGY01000052.1x) and putative non-tardigrade sequences that we removed manually from the final dataset. For example, *Paramacrobiotus richtersi* (Murray, 1911) sequences tPr-orf110, tPr-orf67 and tPr-orf77 exhibited 87%, 87% and 61% identity, respectively, to tubulins in *Nematocida displodere* Luallen, Reinke, Tong, Botts, Félix & Troemel, 2016^40^, a natural microsporidian parasite of *Caenorhabditis elegans*. Notably, the carnivorous *Pam. richtersi* can feed on *C. elegans*, suggesting these sequences are likely contaminations. We also removed several truncated sequences that were identical to longer protein sequences from the same taxa present in the dataset (Supplementary Table S1).

**Figure 2:**
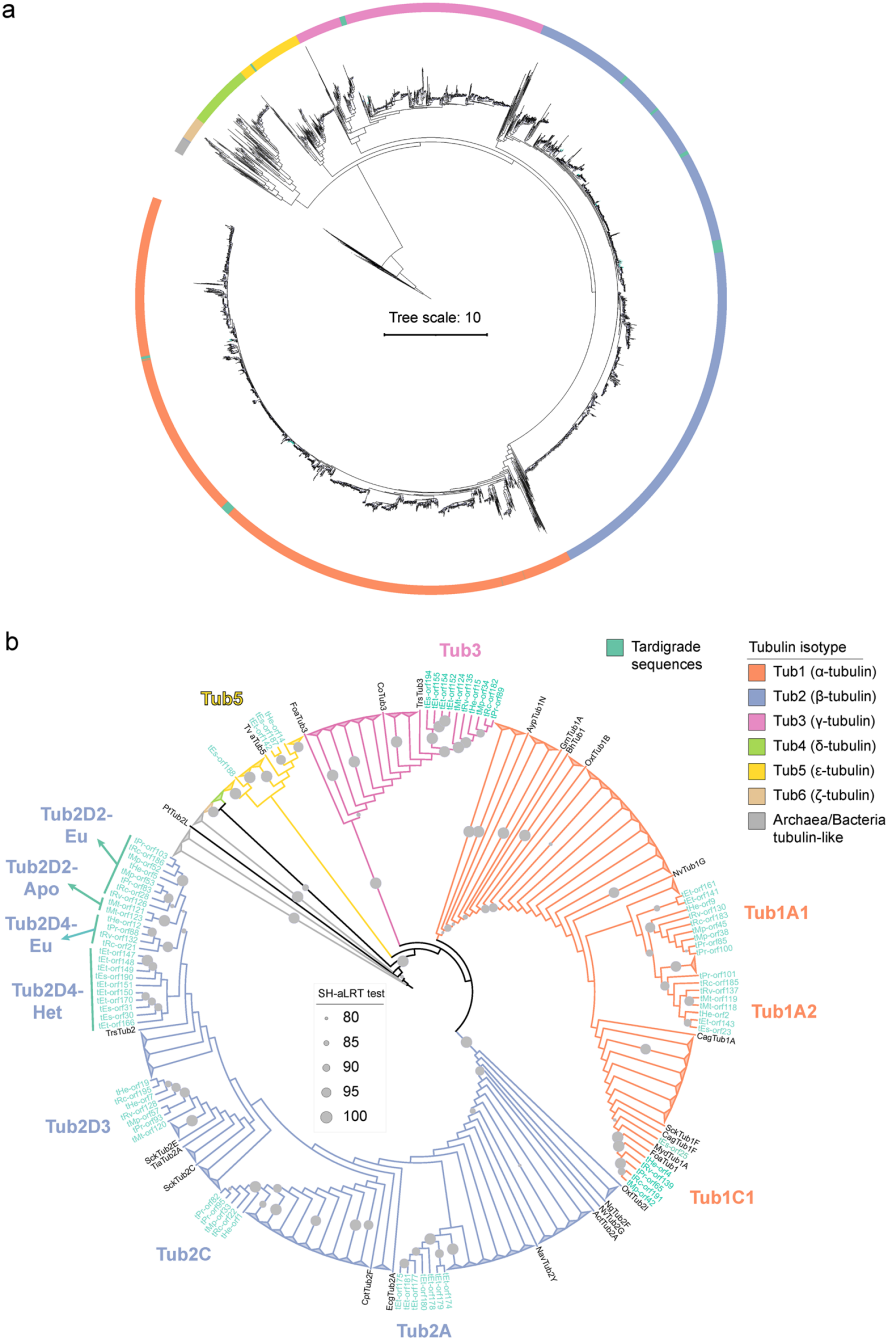
Identification of tardigrade tubulin isoforms. (**a, b**) Different visualizations of an identical phylogenetic tree computed from an alignment of the curated tardigrade tubulin dataset with Findeisen et al.^39^ dataset. (**a**) Maximum likelihood method (IQ-TREE). (**b**) Maximum likelihood method (IQ-TREE), non-tardigrade tubulins branches collapsed and branch lengths ignored to simplify visualization. Grey circles indicate SH-aLRT test score^69^. The legend describes color coding. See also Supplementary data 3 and Supplementary Table 1.

The curated final dataset was aligned to Findeisen et al.^39^ tubulin dataset using the regressive mode of T-Coffee^38^. This alignment served as a template for the computation of the final phylogenetic tree (Fig. 2a, Supplementary Data 3) using the maximum-likelihood method (IQ-TREE). The tardigrade tubulins clustered into α-, β-, γ-, and ε-tubulin isotypes as before. Moreover, we were able to distinguish individual isoforms inside the tubulin isotype clusters based on detailed manual sequence analyses (Fig. 2b, Supplementary Data 3).

In total, we found three α-, seven β-, one γ-, and one ε-tubulin isoforms (Supplementary Table S1). Based on sequence analysis, we suspect that tEs-orf27 might be another example of contamination. Its sequence exhibited 75% identity to *Pollicipes pollicipes*, a barnacle where *Echiniscoides s. sigismundi* (M. Schultze, 1865) is often collected^41^.

**Figure 3:**
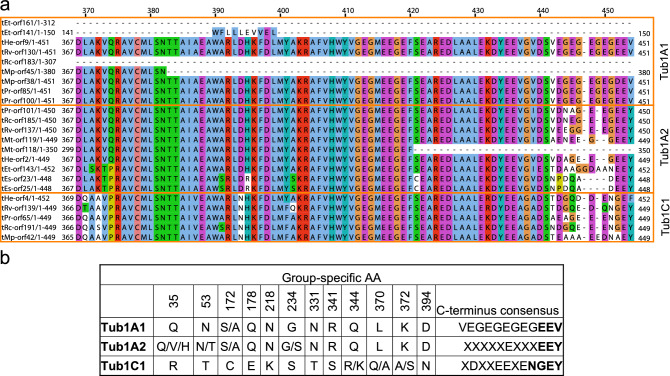
Specific sequence signatures of tardigrade α-tubulins. (**a**) An alignment of the C-terminal region of tardigrade α-tubulins. Orange boxes indicate individual isoforms. Note the very C-terminus. (**b**) Specific AA differences among tardigrade α-tubulin isoforms. See also Supplementary data 4.

We determined three α-tubulin isoforms that we named Tub1A1, Tub1A2, and Tub1C1. In Tub1A1, the C-terminal tyrosine residue is replaced by valine; the Tub1A2 seems to have a relatively similar C-terminus to vertebrate α-tubulins; and the Tub1C1 possesses a C-terminal tyrosine or rarely phenylalanine, but lacks the conserved previous double glutamate (Fig. 3, Supplementary Data 4).

**Figure 4:**
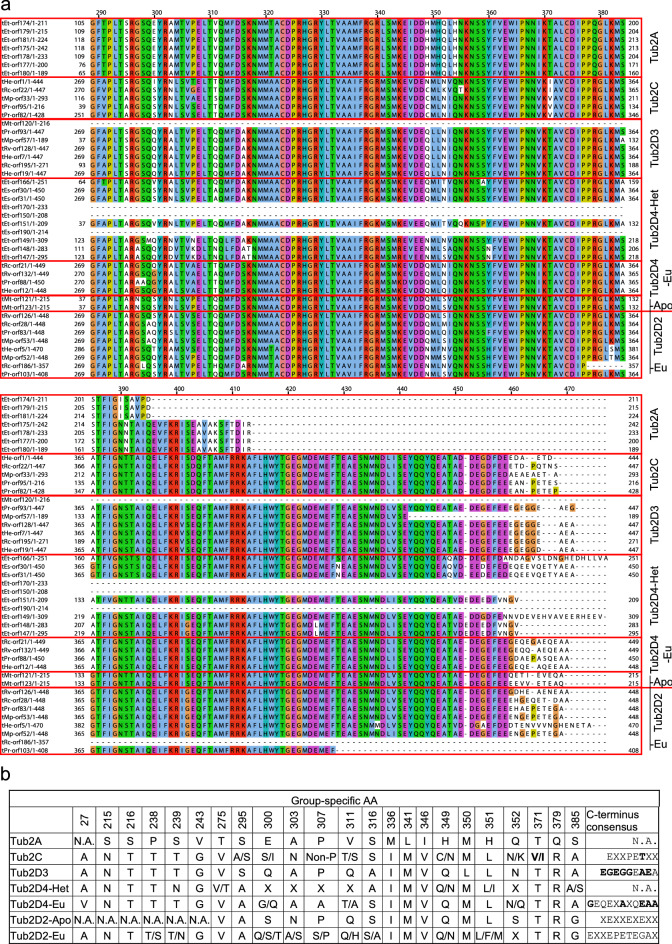
Specific sequence signatures of tardigrade β-tubulins. (**a**) An alignment of the C-terminal region of tardigrade β-tubulins. Red boxes indicate individual isoforms. (**b**) Specific AA differences among tardigrade β-tubulin isoforms. See also Supplementary data 4.

We found seven β-tubulin isoforms with relatively diverse C-termini compared to α-tubulins (Fig. 4, Supplementary Data 4). While the Tub2A isoform from *Echiniscus testudo* (Doyère, 1840) is distant from other tardigrade β-tubulin isoforms, the others share a relatively high degree of similarity, including the heterotardigrade Tub2D4 isoform from *Ech. testudo* and from *Ecn. s. sigismundi*. A conserved hydrophobic amino acid (AA) at the relative position 371 and alanine at the relative position 295 in the Tub2C isoform are interesting. Non-polar AAs at these positions are conserved in many βIII-tubulins in vertebrates and some invertebrates^42,43^. However, other conserved vertebrate βIII-tubulin AAs are absent in Tub2C (Supplementary Data 4).

The γ- and ε-tubulins were found as single isoforms in analyzed tardigrade genomes and transcriptomes (Supplementary Data 4) with two exceptions. However, the truncated version of the *Ech. testudo* γ-tubulin (tEt-orf155, 304 AA) translated in silico from an alternative start codon might correspond to the full-length *Ech. testudo* γ-tubulin (tEt-orf155, 526 AA). Two ε-tubulin sequences from *Ecn. s. sigismundi*—orf187 and orf188 (Supplementary Data 4)—also seem to be fragments of one ε-tubulin sequence, corresponding to N- and C-terminal parts, respectively. However, this must be proven by direct isolation and sequencing of the CDS from *Ecn. s. sigismundi* specimens in the future.

To verify the function of found tubulin sequences we isolated the identified tubulin CDS from tardigrades and expressed them in mammalian cells (for the lack of robust molecular tools for direct expression in tardigrades). We isolated nine out of ten annotated tubulins from *Hypsibius exemplaris* Gąsiorek, Stec, Morek & Michalczyk, 2018^44^ (Supplementary Table S2). The Tub2D5 (Genbank OWA50044.1), which we could not amplify despite trying different primer pairs (Supplementary Table S3), was the exception. Translated protein sequences from isolated tubulin CDS were almost identical to previously annotated versions—except for γ-tubulin—with few single AA changes reflecting likely intra-species diversity (Supplementary Table S2).

Interestingly, the length of the previously annotated γ-tubulin was 912 AA (Genbank OQV14445.1; Supplementary Table S2). In the large dataset that includes thousands of eukaryotic tubulins^39^ and other tardigrade γ-tubulins, no γ-tubulins of similar length were found. We suspected a probable mistake in assigning exon–intron boundaries and thus analyzed the whole coding sequence in the contig (Genbank MTYJ01000105.1). Sequence analysis using NetGene2^45,46^, which predicts splice sites, and *C.* *elegans* settings (*C. elegans* belongs to Ecdysozoa as tardigrades) revealed that the putative donor splice site at the boundary of the exon 4 was dubious. The NetGene2 predicted an alternative splice site further downstream. We modified the coding sequence by adding the putative intronic sequence following the annotated exon 4 until the newly predicted donor splice site (CTGAGCAAAG). This modification gave a stop codon in frame with the coding sequence. Based on this adjustment, we designed a reverse primer downstream of the stop codon (Supplementary Table S3) and successfully amplified a functional γ-tubulin from the adult *Hys. exemplaris* cDNA.

**Figure 5:**
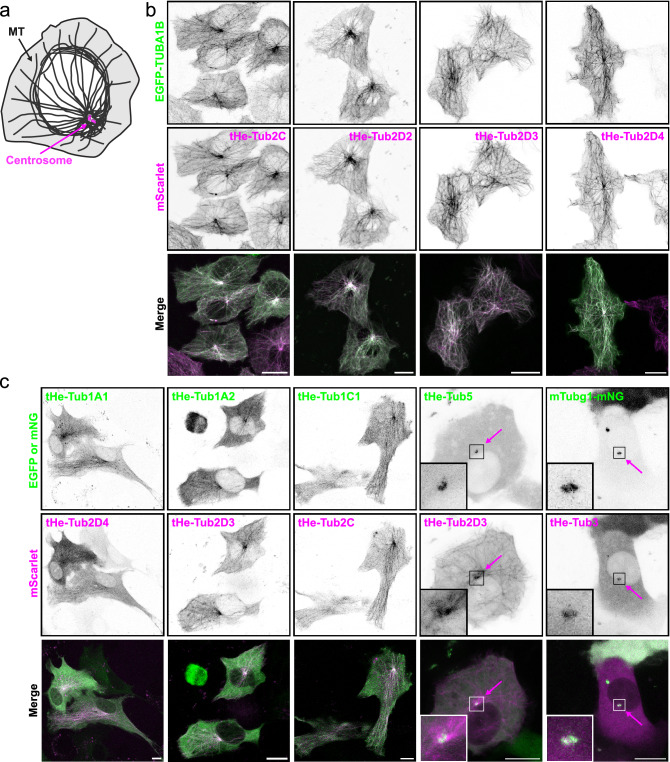
Tardigrade tubulins localize to microtubules or to the centrosomes in mammalian cultured cells. (**a**) A schematic of a mammalian cell with microtubules and the centrosome. (**b**) Maximum intensity projections (MIP) of living U87-MG cells overexpressing indicated constructs. Human EGFP-tagged α-tubulin (TUBA1B) used as a control. Note that all tardigrade β-tubulins localize to microtubules. (**c**) MIP of living hTert-RPE-1 cells overexpressing indicated constructs. Note that tardigrade γ- and ε-tubulins localize to the centrosome (magenta arrows). Scale bars 10 μm.

All tardigrade β-tubulin isoforms tagged with mScarlet fluorescent protein localized properly to microtubules (Fig. 5a, b) labelled with human α-tubulin (EGFP-TUBA1B). The mEGFP-tagged tardigrade α-tubulins localized to microtubules as well, as verified by coexpression with tardigrade β-tubulins (Fig. 5c). In general, tardigrade β-tubulins were more easily detected on mammalian microtubules than tardigrade α-tubulins. The weakest microtubule localization was observed with the tHe-Tub1A2. This tardigrade tubulin was mainly cytoplasmic; however, some microtubule localization was detectable (Fig. 5c). The expected subcellular localization was also observed for tardigrade γ- and ε-tubulins, which localized to the centrosomes in human hTert-RPE-1 cells (Fig. 5c). To conclude, all isolated tardigrade tubulins localized to expected subcellular locations.

## Discussion

We found in this study that tardigrades possess four tubulin subfamilies that function similarly to their vertebrate counterparts. Identifying functional ε-tubulin in the eutardigrade *Hys. exemplaris* and its presence also in heterotardigrades argue in favor of the phylogenetic placement of all tardigrades among Panarthropoda.

Eukaryotic tubulins belong to six major subfamilies (α, β, γ, δ, ε, and ζ), and the last common ancestor of Eukaryota probably had all six tubulin isotypes^39,47^. We found 23 α-, 43 β-, 9 γ-, and 3 ε-tubulin sequences in tardigrades. The number of β-tubulins is generally higher than α-tubulins^39,48^, also confirmed in tardigrades. Notably, tubulin sequences from *Ech. testudo* and *Milnesium t. tardigradum* Doyère, 1840 (newly *Milnesium inceptum* Morek, Suzuki, Schill, Georgiev, Yankova, Marley & Michalczyk, 2019^49^) were mostly truncated, which might reflect the poorer quality of the available sequencing data. Other tardigrade tubulin proteins could still be found with improved transcriptomic, genomic, and proteomic approaches in the future, especially in the abovementioned species.

We found three tardigrade α-tubulin isoforms: Tub1A1, Tub1A2 and Tub1C1. The Tub1C1 was likely present in the ancient Protostomia since its group contains a mollusk Tub1C. The Tub1C1 might have gotten lost in heterotardigrades; however, fragmented and truncated tubulin sequences found in analyzed heterotardigrade transcriptomes make it hard to draw a more definitive conclusion. The Tub1A1 and Tub1A2 form a sister group to Tub1C1 and seem present only in tardigrades. This cluster comprises both Hetero- and Eutardigrada sequences indicating that these isoforms arose by duplication in the last common tardigrade ancestor.

The isoform Tub1A1 has no C-terminal tyrosine residue—its C-terminal AA motif is "EEV." The Tub1C1 possesses a C-terminal tyrosine or rarely phenylalanine; however, it lacks the conserved predeceasing double glutamate—its C-terminal AA motif is "NGEY/F." The question is whether Tub1A1 and Tub1C1 can undergo the classical tyrosination and detyrosination cycle that is typical for most of α-tubulins across many species^29^. On the other hand, all found tardigrade α-tubulins possess a lysine residue equivalent to vertebrate Lys40 that is a target for acetylation^29^. Indeed, an anti-acetylated tubulin antibody was successfully used in studies on tardigrade nervous system^21,31^.

Tardigrade β-tubulin sequences formed seven clusters, many reflecting the class or order distinction of respective taxa. The Tub2D2s from the order Apochela (Tub2D2-Apo) were separated from the other eutardigrades (Tub2D2-Eu). The Tub2D4 sequences were split based on the membership to the class Eutardigrada or Heterotardigrada, in Tub2D4-Eu and Tub2D4-Het, respectively. The Tub2D3, Tub2C and Tub2A formed independent clusters, with the Tub2A being the most distant. Although all sequences in this cluster are truncated, their sequence signature is distinct from the other clusters, justifying the existence of this *Ech. testudo*-specific isoform. C-termini of tardigrade β-tubulins are more diverse compared to α-tubulins, and finding some conserved sequences was difficult.

An interesting isoform is the Tub2C, which might be a homolog of vertebrate βIII-tubulins expressed primarily in neurons and in certain cancer cells^42^. We base our hypothesis on the presence of a hydrophobic AA at the relative position 371 and an alanine 295 in our alignment (designated as AA351 and AA275, respectively, in the literature on vertebrate tubulins^42^). The change from a polar threonine to a non-polar isoleucine or valine at the position equivalent to the vertebrate AA351 is conserved in many βIII-tubulins in vertebrates and even invertebrates^42,43^. Interestingly, the highly extremotolerant *Ramazzottius varieornatus* Bertolani & Kinchin, 1993^50^ lacks the Tub2C isoform and the ε-tubulin as well^51^. These findings imply that the function of Tub2C must be investigated directly in tardigrades in the future.

We wanted to isolate the CDS of tardigrade tubulins directly from tardigrades and test them in cells to verify their function. All tardigrade tubulins tagged with fluorescent proteins (mScarlet or mEGFP) correctly localized to predicted subcellular locations in mammalian cells. All β-tubulins were well incorporated into mammalian microtubules, and their presence was always easily detectable. On the contrary, tardigrade α-tubulins localized to microtubules relatively poorly. The best incorporation was detected for Tub1A1, and the worst microtubule incorporation was observed for Tub1A2. This tubulin exhibited mostly cytoplasmic localization; however, some microtubular localization was also detectable. The Tub1C1 also appeared frequently cytoplasmic; however, microtubule localization became apparent after a longer expression (> 48 h).

Differences in the ability of mEGFP-tagged α-tubulins to incorporate into mammalian microtubules were surprising. It should be noted that the rest of the plasmid backbone, including the linker between the tubulin CDS and the fluorescent protein CDS, was identical. One of the possible explanations for the differences might be the heterogeneity of C-termini, which might impact tubulin heterodimer assembly or incorporation into microtubules. Since we expressed tardigrade tubulins in mammalian cells, an aberrant interaction with some tubulin interactors might have occurred, for instance, with the endogenous tubulin tyrosine ligase (TTL)^52,53^, influencing the incorporation of tardigrade α-tubulins—especially Tub1A2—to microtubules.

The different abilities of individual tardigrade α-tubulins to incorporate into microtubules could also be influenced by temperature. The optimal temperature for *Hys. exemplaris* is between 10 and 18 °C and temperatures above 22 °C are already critical for survival^54^. In contrast, the optimal temperature for mammalian cells used here is 37 °C. Generally, suboptimal temperature causes a decrease in rates of microtubule polymerization and depolymerization, the amount of polymer assembled before catastrophes, and the frequency of microtubule nucleation^55^. However, it is interesting that tardigrade β-tubulins were unaffected, and this phenomenon warrants further investigation, ideally directly in tardigrades.

The ε-tubulin has been identified in all eukaryotic kingdoms but has been lost independently in many clades several times^39^. Remarkably, our data do not support the "ZED module" hypothesis stating that: "(1) organisms that lack epsilon-tubulin always also lack delta- and zeta-tubulin and (2) organisms that have epsilon-tubulin always also have delta- and/or zeta-tubulin^56^." Notably, the ZED module hypothesis was based on a much smaller dataset than that of Findeisen et al.^39^, which we used as a reference in our study.

Currently, tardigrades are classified in the clade Panarthropoda, a part of the superphylum Ecdysozoa. However, the phylogenetic position of tardigrades within the Ecdysozoa remains controversial and debated^20–24,57^. Although tubulins are generally unsuitable for inference of species' phylogenetic position due to the high degree of tubulin gene duplications—especially α- and β-tubulin—occurring spontaneously and independently throughout the evolution^39^, our analysis might still contribute to the refinement of the phylogenetic position of Tardigrada. All Ecdysozoa lack ζ-tubulin^39^. Remarkably, Nematoda also lost their δ- and ε-tubulins; however, some groups of Arthropoda still possess them, including Hymenoptera^39^. We found three unique tardigrade ε-tubulin sequences, two in heterotardigrades and one in the eutardigrade *Hys. exemplaris*. Hence, our data indicate that the current placement of tardigrades among Panarthropoda is correct.

Interestingly, δ-, ε- or ζ-tubulins are not essential for functional cilia in general since there are ciliated species lacking all these isotypes^39^. However, these tubulins are found in species that possess cilia, where they play roles in ciliary assembly and function^39,56,58,59^. Indeed, tardigrades possess cilia that fulfill important sensory tasks^2^. Investigation of the role of tardigrade ε-tubulin for tardigrade ciliary functions will be an exciting avenue of future research. Moreover, our work opens the way for exploration of the tardigrade tubulin code^29,60^ and its role in the fascinating survival abilities of tardigrades.

## Methods

### Identification of tardigrade tubulin sequences

We collected tubulin protein sequences of four model organisms *Homo sapiens, Drosophila melanogaster, C. elegans and Mus musculus* from NCBI. We also acquired published genomes and transcriptomes of eight tardigrade taxa—*Ecn.* cf. *sigismundi*^61^, *Richtersius* cf. *coronifer*^61^, *Ech. testudo*^62^, *Mesobiotus philippinicus* Mapalo, Stec, Mirano-Bascos & Michalczyk, 2016 ^62,63^, *Milnesium tardigradum* (Genbank GFGZ00000000.1), *Pam. richtersi* (Genbank GFGY00000000.1), *Hys. dujardini* (Doyère, 1840) (now *Hys. exemplaris*) (*Hys. dujardini* strain: Z151 Genome sequencing and assembly; (Yoshida et al.^24^) and *Ram. varieornatus* (*Ram. varieornatus genome*, (Hashimoto et al.^51^) (Table 1). Of note, originally, we used genome assemblies of those last two strains from the website tardigrades.org, which has been discontinued. Regarding name abbreviations of tardigrade species/taxa, we followed Perry et al.^64^.

By using the BLAST + software (Version 2.2.26+)^65^, we prepared our local BLAST + database from the abovementioned data. We ran a TBLASTN search in the local tardigrade BLAST + database with an e-value cutoff of 1E10^–5^ using tubulin protein sequences from *H. sapiens*, *M. musculus*, *D. melanogaster* and *C. elegans* as a query (Supplementary Data 5). Multiplicate redundant sequences were filtered out based on unique sequence IDs. Hits with a unique sequence ID were further aligned using Clustal Omega^66^ and identical redundant sequences from the same species were removed. The resulting dataset of about 90 putative tardigrade tubulin sequences was transferred to Benchling and translated based on coordinates from the BLAST + search (frame and strand) (UJEP dataset).

Alternatively, the unique sequences were translated using the esl-translate function of HMMER package (3.1b2 February 2015) and an in-house macro for trimming erroneous N-terminal AA extensions arising from artificial translation of 5' segments of identified transcripts (CRG dataset). To extract the domain sequences of the tardigrade tubulin proteins, we used hmmscan from the HMMER package with the default parameters to scan Pfam32.0 ^67^ and retrieved the hits corresponding to the Pfam tubulin family (PF00091.25) (CRG tubulin domains dataset).

### Multiple Sequence Alignments computation and phylogenetic inference

The phylogenetic analysis was carried out in two steps. First, we computed multiple sequence alignments of the found putative tardigrade tubulin protein or domain sequences together with a previously published large tubulin protein dataset^39^ relying on the regressive mode of TCoffee^38^ (Version_13.45.58.c355d11 with Clustal Omega^66^ guide trees and MAFFT-G-INS-i alignments^68^).

Second, the produced alignments were used as input for phylogenetic reconstruction tools based on maximum likelihood (IQ-TREE^69^, version 2.1.2) and minimum evolution (FastME^70^, version 2.1.6.1).

### Culture of *Hys. exemplaris*

Samples of *Hys. exemplaris* were obtained from envirocom (Tübingen, Germany) and from our own culture. Eutardigrade *Hys. exemplaris* is a freshwater, herbivorous species that typically feeds on algae and was cultured as described previously^54^, but with small modifications. Briefly, tardigrades were cultured on 90 mm plastic Petri dishes. The bottom of all dishes was scratched with a fine sandpaper to allow easier locomotion of tardigrades. Each dish contained 10 mL of Chalkley's medium (0.004 g/L KCl, 0.1 g/L NaCl, 0.006 g/L CaCl_2_, 20 ml/L soil extract; Soil extract was an autoclaved supernatant of fertile, humus-enriched soil and distilled water in 1:2 ratio. The extract was allowed to settle over a few days and then filtered) mixed with 50 mL of spring water (Rajec, Czech Republic). Cultures were fed with green algae *Chlorococcum* sp. by adding ca. one volume concentrated algal culture to four volumes of the tardigrade culture. Each culture was checked every 2 to 3 days with a microscope to ensure tardigrades flourished and the oxygen supply was provided by pipetting. According to need, tardigrades were fed the algae every one or two weeks. During the cleaning/checking procedures, a thin iridescent film on the surface of the medium, dead animals, old exuviae, and different organic residues were removed using a pipette. The 3⁄4 volume of the medium were changed once every ten days. This approach has proven successful in that not all food was consumed and the medium was still fresh. In each Petri dish, the population numbered around 500 individuals. If the number of specimens became too large, juveniles and eggs were transferred to a new culture dish. Cultures were stored in a Q-Cell incubator equipped with a combined cooling and heating system with forced air circulation. The temperature was constantly set at 18.7 °C. The external light source (INVITAL LED) with a combination of white and blue LED was installed into the incubator with a 10 h/14 h (light/dark) photoperiod. Algal culture media have several components in common—sources of nitrogen, phosphorus, vitamins and trace metals. For the culture of *Chlorococcum* sp., we used Bold’s Basal Medium^71^ as a basis. The algae were grown in 250 mL Erlenmeyer flasks with the mouth covered by two layers of parafilm. The algae settled at the bottom, therefore, they were shaken by hand once a week. Flasks were located in the laboratory out of the direct sunlight. The laboratory room temperatures fluctuated between 20 and 24 °C.

### Sample collection and mRNA isolation from *Hys. exemplaris* specimens

We collected adult specimens (ca. 200 μm to 300 μm in length) in their culture medium and removed various contaminating substances using a pipette. Specimens were washed several times, first with distilled water and then with ultrapure water. Tardigrades (ca. 2000 individuals) were placed in a low-retention 1.5 mL microcentrifuge tube. The tube was centrifuged at 10,000 × *g* for 1 min at room temperature. During centrifugation, tardigrades sedimented at the bottom of the tube and compacted into a visible pellet. The supernatant was carefully removed as much as possible without disrupting the pellet. In some cases, samples were flash frozen in liquid nitrogen at this stage. Otherwise, we were quickly crushing the pellet with a sterile plastic pestle for 3 s at room temperature and then resuspend it by adding 200 μL of RLT buffer from the RNeasy Mini Kit (74104, Qiagen) that contained 1% (v/v) 2-mercaptoethanol. Then, we crushed the sample again for 5 s and rapidly froze it in liquid nitrogen. As a next step, we removed the tube from the liquid nitrogen and allowed the sample to thaw. As the sample started to thaw, we were crushing it again for 10 s. We repeated the freeze/thaw/crushing steps five times (until homogenization). At the end, we washed the pestle with 150 μL RLT buffer with 2-mercaptoethanol. For sample homogenization, we mixed the sample five times with a 20 G syringe. We used RNeasy Mini Kit for total RNA isolation according to manufacturer's instructions. The RNA was eluted with RNase-free water. The concentration was measured by UV/VIS spectrophotometer Denovix. RNA was stored at − 80 °C.

### Reverse transcription

The RNA template was converted into cDNA by SuperScript® IV Reverse Transcriptase (18090010, Thermo Fisher Scientific) using oligo dT primers according to manufacturer's instructions. We used RNaseH (M0297S, NEB) to remove RNA duplexed with the newly synthetized cDNA. The cDNA was stored at − 20 °C.

### Cloning of tardigrade tubulin coding sequences

Individual tardigrade tubulin CDS were isolated by PCR using specific forward (fwd) and reverse (rev) primers (Supplementary Table S3) and cDNA from adult *Hys. exemplaris* specimens as a template. The PCR products were purified on an agarose gel and cloned into the pJet1.2 (CloneJet kit, K1231, Thermo Fisher Scientific). To tag tardigrade tubulin CDS with fluorescent proteins, we employed NEBuilder® HiFi DNA Assembly Cloning Kit (E2621L, NEB).

Tardigrade β-tubulins and γ-tubulin were tagged with mScarlet. Corresponding tubulin CDS were amplified from above described pJet1.2-tubulin vectors by PCR using specific primers (Supplementary Table S3, designated as "HiFi"). The pLifeAct_mScarlet-i_N1 (a gift from Dorus Gadella^72^; Addgene plasmid # 85,056; http://n2t.net/addgene:85056; RRID:Addgene_85056) was linearized by NheI and BamHI, which led to the removal of the LifeAct CDS. The linearized recipient plasmid and individual tubulin CDS were assembled using HiFi DNA Assembly creating fusion CDS of tubulins tagged on their C-terminus with mScarlet. The newly characterized *Hys. exemplaris* γ-tubulin obtained Genbank accession number OQ134936.

Tardigrade α-tubulins and ε-tubulin were tagged with mEGFP. First, mEGFP was amplified by PCR using specific primers (Supplementary Table S3, designated as "HiFi") for each tubulin CDS from pGEMHE-NLS-mEGFP (a gift from Melina Schuh^73^; Addgene plasmid # 105,527; http://n2t.net/addgene:105527; RRID:Addgene_105527). Second, each tubulin CDS was amplified by PCR using specific primers (Supplementary Table S3, designated as "HiFi") from above described pJet1.2-tubulin vectors. Third, pEGFP-C1 (Clontech) was linearized by NheI and HindIII which resulted in the removal of EGFP CDS. Finally, recipient plasmid backbone, mEGFP CDS and corresponding tubulin CDS were assembled using HiFi DNA Assembly creating fusion CDS of tubulins tagged on their N-terminus with mEGFP. All plasmids were verified by sequencing. pEGFP-Tub (encoding EGFP-TUBA1B) was originally from Clontech.

### Transfection and confocal microscopy

The hTert-RPE1 and U87-MG cells were maintained in Dulbecco’s Modified Eagle’s Medium (DMEM) or Eagle’s minimal essential medium (E-MEM), respectively, supplemented with 10% (v/v) fetal bovine serum and 0.1% (w/v) penicillin and 0.1% (w/v) streptomycin. Cells were maintained in culture flasks (Falcon or Corning) at 37 °C and 5% CO_2_ in a humid atmosphere in an incubator and subcultured (passaged) every 2 to 3 days after obtaining 80% to 90% confluence. Cells were seeded on 18-well glass-bottom dishes (ibidi) one day before transfection and then transfected using Lipofectamine 3000 (Thermo) or JetOptimus (Polyplus) according to manufacturer’s instructions. Cells were incubated in the incubator and visualized 24 h to 48 h post transfection.

Transfected cells were imaged using a Leica SP8 confocal microscope enclosed in an environmental chamber with constant temperature (36.9 °C), humidity (95%) and 5% CO2 levels. Images were acquired using a 63x/1.40 N.A. oil objective. Filter and detector settings were optimised to minimise any possible bleed-through between fluorescence channels.

Fluorescence images were adjusted in Fiji^74^. All acquired z-stack were processed using Maximum intensity projection and Brightness/Contrast function. Only the Tub1C1 was additionally processed using Substract Background function with 150 pixels rolling ball radius.

## Supplementary Information

Supplementary Information.

## Data Availability

Further information and requests for resources and reagents should be directed to and will be fulfilled by the corresponding author (stanislav.vinopal@ujep.cz). Recombinant DNA generated in this study is available upon request from the corresponding author. The datasets generated and/or analysed during the current study are included in this published article (and its Supplementary Information files) and are also available in the repository Zenodo (https://doi.org/10.5281/zenodo.7528263). All sequenced tubulin CDS of *Hys. exemplaris* were uploaded to GenBank (tHe-Tub1A1, OQ282841; tHe-Tub1A2, OQ282842; tHe-Tub1C1, OQ282843; tHe-Tub2C, OQ282844; tHe-Tub2D2, OQ282845; tHe-Tub2D3, OQ282846; tHe-Tub2D4, OQ282847; tHe-Tub3, OQ134936; tHe-Tub5, OQ282848).
