# Supplementary material for: Phylogenetic and functional characterization of water bears (Tardigrada) tubulins
Source: Sci Rep. 2023 Mar 30;13:5194. doi: 10.1038/s41598-023-31992-z (PMC10063605; doi:10.1038/s41598-023-31992-z)
Supplement: Supplementary file 1 — Supplementary Information. [file 41598_2023_31992_MOESM1_ESM.zip › Novotna Floriancicova_Supplementary data/Supplementary Table S2.docx]

**Supplementary Table S2. Nomenclature of tubulin CDS isolated from *Hys. exemplaris***

| **Tubulin CDS identified in this work (Genbank)** | **Previously identified tubulin CDS (Genbank)** | **Locus tag** | **Abbreviation in alignments** | **Proposed name** |
| --- | --- | --- | --- | --- |
| OQ282841 | OQV18512.1 | BV898_07340 | tHe-orf9 | tHe-Tub1A1 |
| OQ282842 | OQV22293.1 | BV898_03793 | tHe-orf2 | tHe-Tub1A2 |
| OQ282843 | OQV22294.1 | BV898_03794 | tHe-orf4 | tHe-Tub1C1 |
| OQ282844 | OQV22425.1 | BV898_03597 | tHe-orf1 | tHe-Tub2C |
| OQ282845 | OQV19177.1 | BV898_06814 | tHe-orf5 | tHe-Tub2D2 |
| OQ282846 | OQV19199.1 | BV898_06836 | tHe-orf7 | tHe-Tub2D3 |
| OQ282847 | OQV16248.1 | BV898_09557 | tHe-orf12 | tHe-Tub2D4 |
| N/A | OWA50044.1 | BV898_14575 | tHe-orf19 | tHe-Tub2D5*** |
| N/A | OQV14445.1 | BV898_11421 | tHe-orf15 - in the initial alignments (**Supplementary data 1**) | -** |
| OQ134936 | N/A | BV898_11421 | tHe-orf15 - in the final alignment (**Supplementary data 3**) | tHe-Tub3*** |
| OQ282848 | OQV14841.1 | BV898_10989 | tHe-orf14 | tHe-Tub5 |

*Isolation unsuccessful form adult *Hys. exemplaris* cDNA

**γ-Tubulin-like sequence, 912 AA, not isolated, likely an annotation error as discussed in the main text

***Sequence encoding a 460 AA long functional γ-tubulin isolated from adult *Hys. exemplaris* cDNA

N/A = not applicable
