# Supplementary material for: Phylogenetic and functional characterization of water bears (Tardigrada) tubulins
Source: Sci Rep. 2023 Mar 30;13:5194. doi: 10.1038/s41598-023-31992-z (PMC10063605; doi:10.1038/s41598-023-31992-z)
Supplement: Supplementary file 1 — Supplementary Information. [file 41598_2023_31992_MOESM1_ESM.zip › Novotna Floriancicova_Supplementary data/Supplementary Table S3.docx]

**Supplementary Table S3. Primer list**

| **Oligonucleotide name** | **Sequence (5'→3')** |
| --- | --- |
| HeTub1A1_fwd | TACGTCTCCTTAGCAGATCCTC |
| HeTub1A1_rev | GTGCGGGGAGATTACTGTAAAG |
| HeTub1A2_fwd | GGAACCTTCTCTCCTGTTTCTG |
| HeTub1A2_rev | GTTTAAGGACGAGAGATTCCCG |
| HeTub1C1_fwd | CCCATACAGCTCACAAGATTCC |
| HeTub1C1_rev | CCACTCGCTTCCCATGATAAAACAAG |
| HeTub2C_fwd | AGCTAAACAAGGTAACCATGAGG |
| HeTub2C_rev | CACTAGTCAGTTTCCGCATCCTC |
| HeTub2D2_fwd | ATGCTCGGACTCTCTACGCCTTC |
| HeTub2D2_rev | TTTCGACCAGCGGTGAAGTTA |
| HeTub2D3_fwd | GCAAGCATGCGTGAAATCGTC |
| HeTub2D3_rev | GGTCCTTCCAACTAAGCCTCAG |
| HeTub2D4_fwd | CGCTTCAGAAACCCTTATCAGC |
| HeTub2D4_rev | AGAGCGAGAAACTAGTCTAAGCA |
| HeTub2D5_fwd | CACTTAGAAGCGTCAGAAAGATGC |
| HeTub2D5_rev | ATGGTCTAAGCGCGGTTCTTATGC |
| HeTub2D5-new-fwd | TAGAAGCGTCAGAAAGATGCGTG |
| HeTub2D5-new-rev | GTCTAAGCGCGGTTCTTATGCCTC |
| HeTub3_fwd | ATGCCTCGCTCCATCATCTCCCTG |
| HeTub3_rev1 | TGGGACGATGTAAAGGGTGAAGG |
| HeTub3_rev2 | CGGTTAGTCTATTTCACTTTCTTGGATCGG |
| HeTub5_fwd | GCAATTCAAATGTCGCATAATGTGGTCATCTC |
| HeTub5_rev | ACTGTCAGCCCGCCTTCACCTTG |
| mEGFP_HiFi_fwd | TTAGTGAACCGTCAGATCCGCTAGCCACCATGGTGAGCAAGGGCGAG |
| mEGFP_Tub1A1_HiFi_rev | TGCATTCACGCACTCGAGATCTGAGTCCGGACTTGTACAGCTCGTCCATGC |
| mEGFP_Tub1A2_HiFi_rev | TGCATTCGCGCACTCGAGATCTGAGTCCGGACTTGTACAGCTCGTCCATGC |
| mEGFP_Tub1C1_HiFi_rev | TACATTCACGCACTCGAGATCTGAGTCCGGACTTGTACAGCTCGTCCATGC |
| mEGFP_Tub5_HiFi_rev | CATTATGCGACACTCGAGATCTGAGTCCGGACTTGTACAGCTCGTCCATGC |
| Tub1A1_HiFi_fwd_new | ATCTCGAGTGCGTGAATGCATCTCAGTC |
| Tub1A1_HiFi_rev_new | CCGTCGACTGCAGAATTCGATTAAACTTCCTCTCCCTC |
| Tub1A2_HiFi_fwd_new | ATCTCGAGTGCGCGAATGCATCTCTATC |
| Tub1A2_HiFi_rev_new | CCGTCGACTGCAGAATTCGATCAATATTCCTCGCCTTC |
| Tub1C1_HiFi_fwd_new | ATCTCGAGTGCGTGAATGTATCTCAATC |
| Tub1C1_HiFi_rev_new | CCGTCGACTGCAGAATTCGATTAGAATTCGCCATTTTCG |
| Tub2C_HiFi_fwd | TTAGTGAACCGTCAGATCCGCTAGCGCCACCATGAGGGAGATTGTCCATC |
| Tub2C_HiFi_rev | CACCATGGTGGCGACCGGTGAACCTCCACCACCTCCGTCAGTTTCCGCATCCTC |
| Tub2D2_HiFi_fwd | TTAGTGAACCGTCAGATCCGCTAGCGCCACCATGCTCGGACTCTCTACG |
| Tub2D2_HiFi_rev | CACCATGGTGGCGACCGGTGAACCTCCACCACCTCCGGCGGTCTCGTTTTCATG |
| Tub2D3_HiFi_fwd | TTAGTGAACCGTCAGATCCGGCCACCATGCGTGAAATCGTCCATTTG |
| Tub2D3_HiFi_rev | CACCATGGTGGCGACCGGTGAACCTCCACCACCTCCAGCCTCAGCTTCTCCACC |
| Tub2D4_HiFi_fwd | TTAGTGAACCGTCAGATCCGCTAGCGCCACCATGCGCGAAATCGTCCACC |
| Tub2D4_HiFi_rev | CACCATGGTGGCGACCGGTGAACCTCCACCACCTCCAGCAGCCTCCTCCTGGGC |
| Tub3_HiFi_fwd | TTAGTGAACCGTCAGATCCGGCCACCATGCCTCGCTCCATCATC |
| Tub3_HiFi_rev | CACCATGGTGGCGACCGGTGAACCTCCACCACCTCCCATCTTCTTACTCTGCAGTTTC |
| Tub5_HiFi_fwd_new | ATCTCGAGTGTCGCATAATGTGGTCATC |
| Tub5_HiFi_rev_new | CCGTCGACTGCAGAATTCGATCAGCCCGCCTTCACCTT |
